# Multimorbidity between Type 2 Diabetes and Depressive Symptoms in Mexico: Prevalence and Associated Factors From the Nationally Representative ENSANUT 2022 Survey

**DOI:** 10.1111/1753-0407.70177

**Published:** 2026-02-04

**Authors:** Ankita Adhikari, Damith Chandrasenage, Suman Prinjha, Gerardo A. Zavala

**Affiliations:** ^1^ Department of Health Sciences University of York York UK

**Keywords:** depression, Mexico, multimorbidity, survey, type 2 diabetes

## Abstract

**Aims:**

Type 2 Diabetes Mellitus (T2DM) and depression are major public health challenges, with Mexico ranking among the countries with the highest prevalence of both. Research into the bidirectional relationship between the two conditions in Mexico is scarse. This study aims to investigate the prevalence of T2DM and depression, the co‐occurrence of these conditions, the strength of their association, and socio‐demographic, and geographical factors contributing to their prevalence in Mexico.

**Methods:**

We used data from the 2022 National Health and Nutrition Survey. T2DM was self‐report. Depression was assessed using the Center for Epidemiological Studies Depression Scale. Descriptive statistics and multivariate logistic regression models were used to explore the bidirectional relationship and associated factors.

**Results:**

A high prevalence of depressive symptoms (16.7%) and Type 2 Diabetes Mellitus (T2DM, 10.9%) was found in a representative sample for the Mexican population (*n* =11 913). Participants with T2DM had higher odds of depressive symptoms (OR:1.78, 95 95% CI: 1.48–2.14), and those with depressive symptoms were also more likely to have T2DM (OR:1.97, 95% CI:1.52–2.54) for ages 20–59, and for ages 60+ (OR: 1.63, 95% CI: 1.27–2.09). Women were more likely to report depression than men (OR:2.14, 95% CI:1.83–2.51), and older adults (60+) had over three times higher odds of depression compared to younger adults (OR:3.53, 95% CI:3.00–4.15). Higher education was protective against both conditions, with individuals having high school or higher education showing lower odds of depression (OR:0.41, 95% CI: 0.31–0.53) and T2DM (OR: 0.52, 95% CI: 0.37–0.74).

**Conclusions:**

Integrated strategies to address the co‐occurrence of T2DM and depression are needed, particularly among vulnerable and older populations.

## Summary

- What is already known on this topic
  ○ Diabetes and depression frequently co‐occur, with people living with diabetes being two to three times more likely to experience depression, particularly in LMICs like Mexico.
  ○ While shared determinants such as age, gender, and socioeconomic status are known to influence both conditions, these determinants interact or differ across countries, and social and geographical contexts.
- What this study adds
  ○ This is the first study to examine the bidirectional relationship between T2DM and depression using a representative sample for the Mexican population from the 2022 ENSANUT survey.
  ○ It reveals a strong mutual association between the two conditions and shows shared social determinants such as age, gender, education, and well‐being.
- How this study might affect research, practice or policy
  ○ Findings support the integration of depression screening into diabetes care and vice versa, especially for older adults and women who are at higher risk.
  ○ The results can guide policy and research to tailor interventions based on local social, economic, and geographic conditions, acknowledging that determinants of multimorbidity are context‐dependent.

## Graphical abstract

Using 2022 ENSANUT data, we found high prevalence of depressive symptoms (16.7%) and T2DM (10.9%) in Mexico, with 4.6% reporting both. Prevalence varied widely across states. T2DM and depression were strongly associated in all age groups, with women and older adults more likely to report depression. Higher education was protective against both conditions.

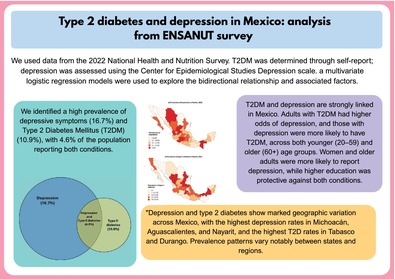

## 1 Introduction

Type 2 diabetes mellitus (T2DM) and depression are both significant global health challenges affecting individuals and health systems [1, 2], with increasing prevalence particularly in low‐and middle‐income countries (LMICs). Significant disparities exist among countries, with India, China and Mexico emerging as the countries with the highest number of new cases [3, 4]. People living with diabetes are two to three times more likely to experience depression compared to the general population [1, 2]. The coexistence of these conditions has severe implications, including poorer clinical outcomes, reduced quality of life, and increased mortality rates [5, 6]. The combination of depression and diabetes also negatively impacts self‐management, glycemic control, and the risk of diabetes‐related complications [7, 8, 9]. This multimorbidity leads to substantially higher healthcare utilization and costs compared to managing diabetes alone [10].

T2D affects over 9% of Mexico's adult population [11], and depression affects approximately 13.6% of Mexican adults, contributing to the mental health burden of the population [12]. Despite the high prevalence of both conditions, research examining their association within Mexico's unique sociocultural and economic context remains limited, with the few available studies focusing on specific population groups. Tovilla‐Zárate et al. (2012) found that nearly half (48%) of outpatients with T2DM in Mexico City experienced depression [13]; and a study on older Mexican Americans reported that T2DM increases the risk of disability and mortality, particularly among those with high depressive symptoms [14].

Previous research using the 2022 ENSANUT survey has concentrated either on aspects of T2D alone, or on mental health alone, with no exploration of their bidirectional relationship. One study, for example, examined four diabetes subgroups across different geographical locations [15], and focused only on the prevalence and regional distribution of diabetes subgroups among different socioeconomic groups. Another study, focusing on depression, explored the prevalence of depressive symptoms among Mexican adolescents and adults using the 2022 ENSANUT survey, reporting an average CESD‐7 score of 3.2 in adolescents and a prevalence of 16.7% in adults, with higher rates observed in older adults, women, those with low wellbeing, and rural residents. None of the 2022 ENSANUT survey studies, however, examined the relationship between T2D and depression. Using the 2018 ENSANUT survey, one study has focused on the association between T2DM and depression but from a syndemic perspective [16] offering a conceptual understanding of their interconnectedness. It found a significant relationship between T2D and depressive symptoms, with obesity, low education, and exposure to violence increasing the risk of co‐occurrence. However, it did not examine how social or regional inequalities shape this relationship. Our study addresses this gap by using the most recent nationally representative data from the 2022 ENSANUT survey to explore in depth how demographic, socioeconomic, and geographical factors contribute to the co‐occurrence of T2D and depression across Mexico. In addition, it provides updated estimates of depression and its associations in the post‐COVID‐19 context, acknowledging how the pandemic may have shaped both the prevalence and determinants of depression [17].

Given the significant burden of diabetes and depression on Mexico's healthcare system and economy, our study aims to investigate the bidirectional relationship between these two chronic conditions, the strength of their association and the socio‐demographic, and geographical factors contributing to theit prevalence using the most recent survey that researchers have access to: the 2022 ENSANUT survey. To our knowledge, this is the first study to explore the bidirectional relationship between T2DM and depression at a population level. This epidemiological analysis will contribute to a deeper understanding of the complex interplay between diabetes and depression in Mexico and can inform the development of effective future interventions [18, 19].

## 2 Research Design and Methods

This study adheres to the Strengthening the Reporting of Observational Studies in Epidemiology (STROBE) guidelines for cross‐sectional studies, ensuring transparent reporting of the methodology, analysis, and findings [20].

### 2.1 Study Design

This study is a secondary data analysis of the latest publicly available 2022 National Health and Nutrition Survey (ENSANUT), a nationally representative, cross‐sectional survey conducted in Mexico [21]. ENSANUT employs a multi‐stage probabilistic sampling method to ensure representativeness at national and regional levels, including rural, urban, and metropolitan areas. Data were collected between July and December 2022, with a response rate of 73%, covering 10 160 households.

Sample weights in ENSANUT were corrected using expected response rates from 2021 and calibrated through post‐stratification adjustments to account for over‐ or underrepresentation of specific subgroups. The final weights allow the sample size to expand to represent the Mexican population accurately, based on household (state, rural/urban/metropolitan area) and individual (age group) characteristics. Participants completed a comprehensive questionnaire that collected demographic, socioeconomic, and health‐related data, alongside physical examinations measuring blood pressure and anthropometry.

### 2.2 Study Population

This article considers information from the health questionnaires of adults (*n* = 11 913).The survey protocol received approval from the Ethics, Research, and Biosecurity Committees of the National Institute of Public Health “(approval number CI: 1807/S7‐2022)”. All participants were informed about the survey's objectives and procedures, and provided written informed consent prior to participation.

### 2.3 Data Collection

Data were collected face‐to‐face as part of the 2022 National Health and Nutrition Survey (ENSANUT). Trained and standardized personnel conducted interviews using structured questionnaires to gather information on demographic, socioeconomic, and health‐related characteristics. Anthropometric measurements, including height and weight, were also obtained during the survey to ensure accurate and reliable health data.

### 2.4 Measures

#### 2.4.1 Type 2 Diabetes

Diabetes status was based on self‐reported physician diagnosis. Participants were asked if a doctor had ever diagnosed them with diabetes, and those who responded affirmatively were categorized as having diabetes.

#### 2.4.2 Depression

Depressive symptoms were assessed using the 7‐item version of the Center for Epidemiological Studies Depression Scale (CES‐D), a validated and widely used tool in population‐based surveys [22]. The CES‐D measures the severity of depressive symptoms over the past two weeks, with scores ranging from 0 to 21. Participants were classified based on their scores: those with ≥ 9 points were identified as having moderate or severe depressive symptoms, while those with ≤ 8 points were considered to have minimal or no symptoms. For older adults (aged 60 and above), the cutoff for moderate or severe depressive symptoms was set at 5 points [23]. This instrument has been validated for screening for the general, older population and for patients with chronic conditions [23, 24]. It evaluates cognitive and behavioral symptoms and is designed to be straightforward to understand and use.

#### 2.4.3 Covariates

We included covariates known from previous studies to influence either diabetes or depressive symptoms. Adjusting for these demographic, socioeconomic, and health‐related factors allows for a more accurate analysis of their relationship while accounting for potential confounding variables.

Demographic variables included age (measured in years) and sex (male/female), as both are well‐established determinants of health, with older adults and women showing higher prevalence rates of both diabetes and depression. Socioeconomic status was assessed using household income, categorized into predefined quintiles, and education level, grouped as primary, secondary, and higher education.

Geographical location was included to account for regional differences in healthcare access and cultural practices. Participants were classified by region (Frontera, Pacific‐Central, Centro‐North, Centro, and others) based on their place of residence.

### 2.5 Statistical Analysis

Descriptive statistics were used to summarize the characteristics of the study population. For categorical variables, frequencies and percentages were reported, while continuous variables were summarized using means and standard deviations. Key demographic, socioeconomic, and health‐related variables were compared across groups to identify patterns and disparities in the study population. This included the distribution of age, sex, education levels, income quintiles, and regional representation. The prevalence of diabetes and depressive symptoms was also calculated and stratified by these variables to provide a detailed population profile.

To investigate the bidirectional relationship between diabetes and depression, two multivariable logistic regression models were constructed: *Model 1*: diabetes was the independent variable, with depression as the dependent variable; and *Model 2*: depression was the independent variable, with diabetes as the dependent variable. Both models were adjusted for potential confounders, including sex, age, education, socioeconomic status, and geographic area. The inclusion of these covariates ensured that the analysis accounted for known factors that could influence the relationship between diabetes and depression. Multicollinearity was evaluated using polychoric correlations among categorical predictors, Pearson correlations among continuous variables, and variance inflation factors (VIFs), with all VIFs < 5. Model fit was evaluated using the Hosmer–Lemeshow test, Nagelkerke pseudo‐R^2^, and the area under the receiver operating characteristic curve (AUC). Results were expressed as odds ratios (OR) with 95% confidence intervals (CI), and statistical significance was set at *p* < 0.05. All statistical analyses were performed using Stata version 18 (StataCorp, College Station, Texas).

## 3 Results

### 3.1 Characteristics of the Population

The study consisted of 11 913 participants representing the full Mexican population, with a mean age of 46.3 years (SD = 16.7) (Table 1). Women comprised 60.4% of the sample, and 76.4% of participants were aged 20–59 years. Depressive symptoms were reported by 19.5% of the population, with prevalence notably higher among those aged 60 years and older (40.9%) compared to the younger age group (12.9%). The prevalence of self‐reported diabetes was 13.0%. The majority of the sample (67.7%) had completed high school or higher education, while 26.6% reported secondary education and 5.8% had below‐secondary education. Socioeconomic status (SES) showed a slight majority from low‐SES households (54.3%). Most participants resided in urban areas (75.8%), with regional representation ranging widely, including 21.9% from Mexico City.

**TABLE 1:** Characteristics of the population.

	Mean	SD	Mean adjusted (thousands)	SD
Depressive symptom score^a^ (mean, SD)	4.06	4.4	85 520 767	4.2
Age in years (mean, SD)	46.36	16.7	85 520 767	16.5

	*N*	%	*N* weighted (thousands)	%
Depression^b^				
No	9586	80.5	71 270 286	83.3
Yes	2327	19.5	14 250 481	16.7
Depression by age group				
Age 20–59				
No	7928	87.1	60 719 248	88.7
Yes	1178	12.9	7 701 384	11.3
Age 60+				
No	1658	59.1	10 551 038	61.7
Yes	1149	40.9	6 549 097	38.3
Diabetes				
No	10 351	86.9	76 122 433	89.0
Yes	1544	13.0	9 282 126	10.9
Missing	18	0.2	116 208	0.1
Sex				
Males	4724	39.7	40 885 126	47.8
Females	7189	60.4	44 635 641	52.2
Age				
20–59	9106	76.4	68 420 632	80.0
60+	2807	23.6	17 100 135	20.0
Education				
Below secondary	690	5.8	4 057 588	4.7
Secondary school	3163	26.6	19 610 660	22.9
High school or above	8060	67.7	61 852 519	72.3
Socioeconomic status (Material of floor)				
low‐SES	6466	54.3	45 439 481	53.1
high‐SES	5428	45.6	39 946 657	46.7
missing	19	0.2	134 629	0.2
Well‐being index				
Low	3934	33.0	25 606 047	29.9
Medium	3983	33.4	27 913 137	32.6
High	3996	33.5	32 001 584	37.4
Area				
Urban	9028	75.8	68 364 850	79.9
Rural	2885	24.2	17 155 917	20.1
Region				
Pacific North	1351	11.3	8 090 857	9.5
Border	2175	18.3	11 087 071	13.0
Pacific‐central	735	6.2	9 293 140	10.9
North centre	2728	22.9	10 753 727	12.6
Centre	829	7.0	8 519 059	10.0
Mexico City	1267	10.6	18 754 320	21.9
South pacific	1244	10.4	10 593 705	12.4
Peninsula	1584	13.3	8 428 889	9.9
Total	11 913	100	85 520 768	100

^a^ Score from the 7‐item version of the Center for Epidemiological Studies Depression Scale (CES‐D).

^b^ CES‐D scores ≥ 9 points.

### 3.2 Depression and Diabetes Distribution in Mexico

**FIGURE 1:**
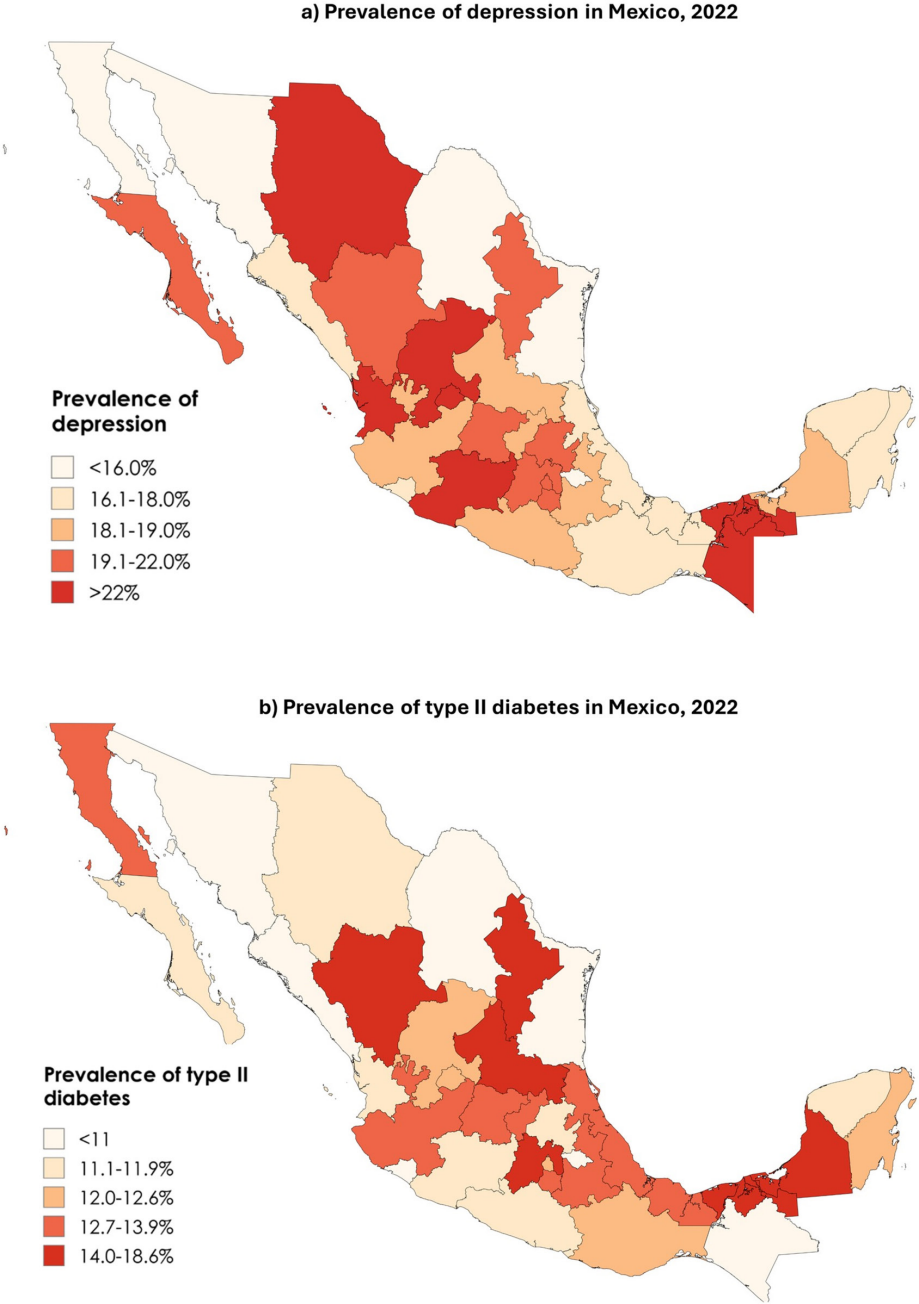
Prevalence of depression and type 2 diabetes across Mexican states.

As seen in Figure 1. The prevalence of both depression and type 2 diabetes varies significantly across Mexican states and regions. At the state level, the highest rates of depression were observed in Michoacán (26.8%), Aguascalientes (26.4%), and Nayarit (25.2%), while the lowest were found in Coahuila (13.0%) and Sonora (13.7%). Regionally, depression prevalence ranged from 15.1% in the Pacific North to 18.7% in the Pacific Central region, with the North‐Central and Peninsula regions also reporting high rates (18.0%). Intermediate rates were found in the Border (16.2%) and México City (16.5%) regions, while slightly lower rates were seen in the Centre (15.4%) and South Pacific (15.3%). Similarly, T2D prevalence ranged from 8.4% in Chiapas to 18.6% in Tabasco and 18.1% in Durango at the state level. At the regional level, prevalence ranged from 9.6% in the Pacific Central to 12.0% in the Centre. The Border and North‐Central regions each reported 11.5%, while the Peninsula (11.0%) and South Pacific (11.1%) regions had slightly lower rates. The Pacific North and México City reported rates of 9.9% and 10.5% respectively.

### 3.3 Diabetes and Depression Multimorbidity

**FIGURE 2:**
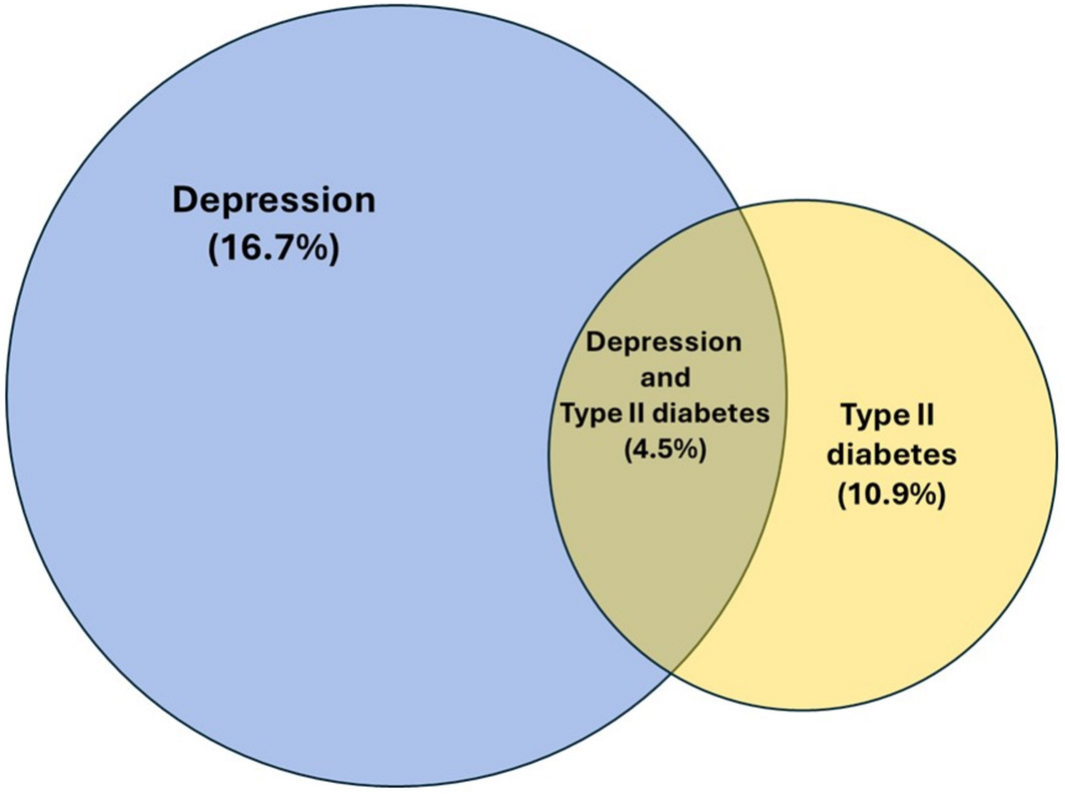
Prevalence of depression, T2D and co‐occurring conditions in Mexico.

As shown in Figure 2, 4.5% of the total Mexican adult population experiences both diabetes and depression multimorbidity. Additionally, one‐third of individuals with depression also have diabetes, while nearly half of those with diabetes also report experiencing depression.

### 3.4 Stratified Depression and Diabetes Analysis

As seen in Table S1, the analysis stratified by depression revealed that 19.5% of participants reported depressive symptoms, with a higher prevalence among individuals with diabetes (34.8%) compared to those without (17.3%). Women were more likely to report depression (24%) than men (12.8%). Depression was also more prevalent among older adults, affecting 40.9% of those aged 60 years or older, compared to 12.9% in the 20–59 age group. Educational attainment showed an inverse relationship with depressive symptoms: 42.3% of participants with below‐secondary education reported depression, compared to 30.1% with secondary education and 13.4% with high school or above. Similarly, individuals with low well‐being levels had the highest prevalence of depression (24.1%), while those with high well‐being levels had the lowest (15.1%). Geographical factors also played a role, with rural residents experiencing slightly higher rates of depression (21.4%) compared to urban residents (18.9%). The highest depression prevalence was observed in Pacific‐Central (21.2%) and the lowest in Mexico City (17.7%).

The analysis stratified by diabetes (Table S2) showed that 13% of participants reported having diabetes, with a higher prevalence observed among individuals with depressive symptoms (32.8%) compared to those without (10.5%). Women had a slightly higher diabetes prevalence (14%) than men (11.4%). Age was a significant factor, with diabetes affecting 28% of those aged 60 years or older compared to 8.3% in the 20–59 age group. Educational attainment showed an inverse relationship with diabetes prevalence, as 24.8% of participants with below‐secondary education reported diabetes, compared to 20.1% with secondary education and 9.2% with high school or above. Socioeconomic status and well‐being indices also influenced diabetes prevalence; individuals with low SES and low well‐being had higher diabetes rates (12.5%) compared to those with high SES (11.4%) and high well‐being (13.3%). The highest prevalence of diabetes was reported in the Pacific‐North (13.3%) and the lowest in Mexico City (10.5%).

### 3.5 Bidirectional Association Between Depression and Diabetes

As shown in Table 2, participants with diabetes had significantly higher odds of reporting depressive symptoms (OR: 1.78, 95% CI: 1.48–2.14). Females were more likely to report experiencing depressive symptoms compared to males (OR: 2.14, 95% CI: 1.83–2.51). Older participants (aged 60+) had higher odds of depression compared to younger adults (aged 20–59) (OR: 3.53, 95% CI: 3.00–4.15). Education showed a protective effect, with those having high school or higher education experiencing reduced odds of depression (OR: 0.41, 95% CI: 0.31–0.53) compared to those with less than secondary education. Additionally, participants with a high well‐being index had decreased odds of depressive symptoms (OR: 0.62, 95% CI: 0.50–0.77). Both logistic regression models demonstrated adequate fit, acceptable discrimination, and minimal multicollinearity among predictors (see Appendix A).

**TABLE 2:** Association between depression, type 2 diabetes and sociodemographic variables.

	OR (95% CI)	
	Unweighted	Weighted
Diabetes		
No (ref.)		
Yes	1.58 (1.39,1.80)	1.78 (1.48,2.14)
Sex		
Males (ref.)		
Females	2.18 (1.96,2.42)	2.14 (1.83,2.51)
Age		
20–59 (ref.)		
60+	3.4 (3.04,3.81)	3.53 (3.00,4.15)
Education		
Below secondary (ref.)		
Secondary school	0.78 (0.65,0.94)	0.63 (0.49,0.82)
High school or above	0.49 (0.40,0.59)	0.41 (0.31,0.53)
Well‐being index		
Low (ref.)		
Medium	0.81 (0.71,0.91)	0.79 (0.65,0.95)
High	0.63 (0.55,0.73)	0.62 (0.50,0.77)
Area		
Urban (ref.)		
Rural	0.89 (0.79,1.01)	1.06 (0.88,1.28)
Region		
Pacific North (ref.)		
Border	1.06 (0.88,1.29)	1.08 (0.85,1.38)
Pacific‐central	1.17 (0.92,1.50)	1.21 (0.85,1.73)
North centre	1.09 (0.91,1.31)	1.11 (0.88,1.41)
Centre	0.85 (0.67,1.09)	0.78 (0.56,1.08)
Mexico City	1.12 (0.90,1.38)	1.05 (0.81,1.37)
South pacific	0.79 (0.63,0.98)	0.74 (0.55,1.00)
Peninsula	1.00 (0.82,1.22)	0.98 (0.75,1.27)

Participants with depressive symptoms had significantly higher odds of reporting diabetes compared to those without (age 20–59: OR: 1.97, 95% CI: 1.52–2.54; age 60+: OR: 1.63, 95% CI: 1.27–2.09), (Table 3). Women had slightly higher odds of diabetes than men, though this association was not significant in the weighted analysis (OR: 1.00, 95% CI: 0.85–1.19). Older participants (60+) were significantly more likely to report diabetes than those aged 20–59 (OR: 3.41, 95% CI: 2.69–4.33). Higher education appeared protective, with participants having high school or above education showing lower odds of diabetes (OR: 0.52, 95% CI: 0.37–0.74) compared to those with below‐secondary education. A higher well‐being index had a higher likelihood of diabetes (OR: 1.55, 95% CI: 1.22–1.98 for high well‐being). Geographic and regional factors were not strongly associated with diabetes, indicating relatively uniform distribution across locations.

**TABLE 3:** Association between type 2 diabetes, depression and sociodemographic variables.

Depression (by age group)	OR (95% CI)	
	Unweighted	Weighted
No (ref.)		
Yes (age 20–59)	1.94 (1.61,2.34)	1.97 (1.52,2.54)
Yes (age 60+)	1.35 (1.14,1.60)	1.63 (1.27,2.09)
Sex		
Males (ref.)		
Females	1.15 (1.02,1.29)	1.00 (0.85,1.19)
Age		
20–59 (ref.)		
60+	3.37 (2.90,3.91)	3.41 (2.69,4.33)
Education		
Below secondary (ref.)		
Secondary school	0.93 (0.76,1.14)	0.87 (0.64,1.19)
High school or above	0.55 (0.44,0.68)	0.52 (0.37,0.74)
Well‐being index		
Low (ref.)		
Medium	1.24 (1.07,1.44)	1.25 (1.00,1.56)
High	1.45 (1.24,1.70)	1.55 (1.22,1.98)
Area		
Urban (ref.)		
Rural	0.87 (0.76,1.01)	0.84 (0.67,1.06)
Region		
Pacific North (ref.)		
Border	1.23 (0.99,1.53)	1.16 (0.89,1.52)
Pacific‐central	1.00 (0.74,1.34)	0.89 (0.59,1.32)
North centre	1.16 (0.94,1.43)	1.15 (0.88,1.50)
Centre	1.12 (0.84,1.48)	1.31 (0.90,1.90)
Mexico City	1.08 (0.85,1.38)	1.04 (0.76,1.41)
South pacific	1.09 (0.85,1.40)	1.16 (0.82,1.65)
Peninsula	1.25 (0.99,1.59)	1.24 (0.92,1.68)

## 4 Discussion

In this large nationally representative cross‐sectional analysis, we found a high prevalence of both depressive symptoms (16.7%) and diabetes (10.9%) in Mexico. We found a strong bidirectional association between the two conditions with individuals with diabetes having 78% higher odds of experiencing depressive symptoms and people with diabetes having double the odds of having depression. Women were more likely to report depression than men, and older adults (60+) had over three times the odds of experiencing depression compared to younger adults (20–59). Education was a protective factor, with individuals completing high school or higher education showing reduced odds of depression and diabetes.

The bidirectional relationship between diabetes and depression observed in this study aligns with global evidence [1, 25], including findings from a pooled analysis showing that the prevalence of depression is twice as high among individuals with diabetes [26] and that the risk of depression is 1.33 times greater in this population [27]. A study by Juárez‐Rojop et al. [28] identified elevated HbA1c levels among Mexican individuals with coexisting depression and diabetes, reporting a depression prevalence of 42.8%. Our findings are also consistent with previous research conducted in other LMICs. For instance, in Ghana, approximately one‐third of patients with diabetes were found to experience depression [29] and in Bangladesh, individuals with diabetes were reported to have a sevenfold higher likelihood of comorbid depression [30]. Our study builds on previous evidence by analyzing the bidirectional relationship between these two conditions within the Mexican context, providing timely epidemiological insights into their distribution, the prevalence of multimorbidity, and the sociodemographic factors associated with both depression and diabetes.

Epidemiological studies have consistently demonstrated a gender disparity in the prevalence of depression, with women reporting approximately twice the lifetime risk compared to men and the higher prevalence of depression among women in our study corroborates existing research [31, 32]. This reflects both biological susceptibilities and sociocultural factors that exacerbate stress and reduce access to mental health resources [31]. Similarly, the protective effect of education and higher SES against depression gives evidence of the critical role of health literacy, economic stability, and access to healthcare in mitigating mental health risks [31]. For women, these factors may be particularly influential in shaping their mental health outcomes [32].

Older age was the primary risk factor for both depression and diabetes, with individuals aged 60 and above showing significantly higher prevalence rates of both conditions [33]. The higher prevalence of depression in older adults with diabetes can be attributed to various factors, including the cumulative burden of chronic diseases, reduced physical functionality, social isolation, and the psychological stress of managing long‐term health conditions [34]. Additionally, older adults may face barriers to accessing mental health resources, such as stigma, lack of mobility, or limited healthcare coverage [34, 35, 36]. These factors can lead to poorer depression outcomes in this population, including increased severity and chronicity of symptoms. Given these challenges, emphasis should be placed on developing integrated care models that address the unique needs of older adults, incorporating mental health support into diabetes care, and creating age‐friendly healthcare environments that promote social engagement and psychological well‐being [35].

While the adjusted models indicate that geographic and regional factors were not strongly associated with depression and T2DM, this finding should be interpreted with caution. The adjusted analysis was conducted at the regional level, using aggregated averages across states in a region rather than more granular state‐level estimates. This approach may smooth over important within‐region variation. For example, as shown in Figure 1, there are substantial differences in T2DM prevalence between individual states, such as Chiapas (8.4%) and Tabasco (18.6%). These differences remain relevant from a public health perspective. They may reflect unmeasured local factors such as disparities in healthcare access, food environments, or health literacy. These factors deserve further investigation through more targeted, state‐level analyses.

Addressing the dual burden of diabetes and depression requires improving living conditions, reducing inequalities, and enhancing access to education and healthcare [37]. Targeted approaches, such as integrating depression screening into diabetes care and vice versa, can help identify and address multimorbidity early. For example, behavioral activation (BA) interventions have shown promise in managing depression in individuals with diabetes, particularly in LMICs [18, 19, 38]. Exercise, dietary interventions, blood glucose monitoring, and antidepressant medications, can also be beneficial, as well as structured diabetes education [39]. Expanding the availability of such interventions, coupled with training for healthcare providers in integrated care approaches, can improve outcomes for affected populations [40].

## 5 Strengths and Limitations

This study utilizes data from the 2022 ENSANUT, a nationally representative survey with a robust sampling design that ensures coverage across various regions and demographic groups in Mexico [20]. The rigorous data collection procedures and quality control measures employed by ENSANUT enhance the reliability and validity of the findings. The timeliness of the data allows for an up‐to‐date assessment of the prevalence and associations between diabetes and depression, providing insights for current public health strategies. Further, to enhance the generalisability of our findings to the broader Mexican population and address potential biases, such as non‐participation bias, we applied weighted estimates using inverse probability of response. However, there are limitations worth noting. The cross‐sectional design precludes the establishment of causal relationships between diabetes and depression, limiting the ability to determine the directionality of associations. T2DM status was based on self‐reported physician diagnosis, which may introduce recall bias or misclassification, as some individuals might inaccurately report their diagnosis or be unaware of an undiagnosed condition. Similarly, depressive symptoms were assessed using the 7‐item CES‐D Scale, a validated screening instrument that captures symptom burden rather than a formal clinical diagnosis. While this tool allows for the identification of individuals at risk of depression in large‐scale population studies, it may affect the precision of prevalence estimates and the strength of associations. These limitations should be considered when interpreting the associations observed in this study. Despite these limitations, the CES‐D has been widely used in national health surveys in the US and UK [41], and in the Korean National Health and Nutrition Examination Survey (KNHANES) [42], supporting its validity in population‐level research. The study's survey was conducted during the COVID‐19 pandemic, a period characterized by reported weight gain, higher sedentary behavior, and heightened stress among individuals with diabetes [42]. Consequently, we cannot exclude the possibility that these factors may have influenced our findings. Furthermore, certain factors that may influence the association, such as genetic predispositions, were not measured, potentially omitting important variables from the analysis.

## 6 Conclusions

Our findings indicate a high prevalence of diabetes and depression in Mexico and significant associations with sociodemographic factors. These factors, particularly given the increasing rates of diabetes in Mexico, necessitate a comprehensive public health response, including efforts to address structural determinants of health while incorporating targeted, evidence‐based interventions into routine healthcare. Future research should explore the lived experience of people with diabetes and depression in Mexico and the codesign of interventions to improve quality of life.

## Author Contributions

Study conception and design: Ankita Adhikari and Gerardo A. Zavala; Analysis and interpretation of results: Damith Chandrasenage and Gerardo A. Zavala; Draft manuscript preparation: Suman Prinjha, Ankita Adhikari and Gerardo A. Zavala.

## Funding

This work was partially supported by the National Institute for Health Research (NIHR), Global Health Research project NIHR200806. The views expressed are those of the authors and not necessarily those of the NIHR or the UK Department of Health and Social Care. The funders had no role in study design, data collection and analysis, decision to publish, or preparation of the manuscript.

## Ethics Statement

The ENSANUT 2022 survey protocol received approval from the Ethics, Research, and Biosecurity Committees of the National Institute of Public Health in Mexico (approval number CI: 1807/S7‐2022). This study is a secondary data analysis of anonymised, publicly available data and did not require additional ethical approval.

## Consent

Written informed consent was obtained from all participants by ENSANUT during data collection. This study involved only secondary analysis of anonymised survey data and did not involve any direct contact with participants.

## Conflicts of Interest

The authors declare no conflicts of interest.

## Supporting information


**Table S1:** Stratified characteristics according to depressive symptoms status.
**Table S2:** Stratified characteristics according to type II diabetes status.

## Data Availability

The data used in this study are publicly available and were obtained from the 2022 National Health and Nutrition Survey (ENSANUT), conducted by the National Institute of Public Health (INSP) in Mexico. Access to the dataset is available via https://ensanut.insp.mx upon request and approval from INSP. No identifiable or sensitive information was used in this analysis.

## Appendix A Goodness of Fit and Multicollinearity of the Logistic Regression Models

### Goodness of Fit of the Models

**Table:**

Statistic	Depression model	Diabetes model
N (observations)	11 895	11 895
LR χ^2^ (df), *p*‐value	1430.5 (15), < 0.001	796.4 (16), < 0.001
AIC (full model vs. null model)	10351.97 vs. 11752.48	8420.85 vs. 9185.20
BIC (full model vs. null model)	10470.12 vs. 11759.87	8546.38 vs. 9192.59
Pseudo‐R^2^ (Nagelkerke R^2^)	0.18	0.12
Hosmer–Lemeshow χ^2^ (df), *p*‐value	16.21 (8), *p* = 0.040	9.50 (8), *p* = 0.30
Brier score	0.34	0.1
AUC (ROC)	0.74	0.71
Overall correct classification (%)	81.2	87

### Multicollinearity Test

#### Variance Inflation Factor (VIF)

**Table:**

Variable	Depression		Diabetes	
	VIF	1/VIF	VIF	1/VIF
1.diabetes	1.08	0.92971	—	—
Depression				
1			1.06	0.943395
2			1.56	0.641602
1.adul_sex	1	0.995274	1.02	0.978108
60.adulage	1.3	0.769773	1.78	0.562992
Education				
1	4.23	0.236385	4.25	0.23537
2	4.95	0.202074	4.98	0.200839
Well‐being index				
2	1.55	0.646007	1.55	0.645847
3	1.78	0.563063	1.78	0.561858
1.area2	1.24	0.807268	1.24	0.806967
Region				
2	2.15	0.465215	2.15	0.465281
3	1.45	0.688232	1.45	0.688106
4	2.36	0.424186	2.36	0.424066
5	1.53	0.654085	1.53	0.654052
6	1.74	0.574153	1.74	0.57375
7	1.77	0.563484	1.78	0.563247
8	1.96	0.510093	1.96	0.51019
Mean VIF	2.01		2.01	

Variance Inflation Factor (VIF) is less than 5.

### Multicollinearity Test

#### Variance Inflation Factor (VIF)

**Table:**

Variable	Depression	Diabetes	Sex (female)	Age category	Education (reduced)	Well‐being index	Area (rural)	Region
Depression	1	0.3	0.26	0.5	−0.37	−0.15	0.05	0.03
Diabetes	0.3	1	0.08	0.46	−0.29	0.02	−0.02	0.01
Sex (female)	0.26	0.08	1	0.05	−0.05	−0.03	0.02	0.03
Age category (60+)	0.5	0.46	0.05	1	−0.62	−0.06	0.05	0.03
Education (reduced)	−0.37	−0.29	−0.05	−0.62	1	0.43	−0.34	−0.14
Well‐being index	−0.15	0.02	−0.03	−0.06	0.43	1	−0.54	−0.29
Area (rural)	0.05	−0.02	0.02	0.05	−0.34	−0.54	1	0.23
Region	0.03	0.01	0.03	0.03	−0.14	−0.29	0.23	1

No extreme (Corr. > 0.80) correlations.
