# Supplementary material for: Multimorbidity between Type 2 Diabetes and Depressive Symptoms in Mexico: Prevalence and Associated Factors From the Nationally Representative ENSANUT 2022 Survey
Source: J Diabetes. 2026 Feb 4;18(2):e70177. doi: 10.1111/1753-0407.70177 (PMC12869113; doi:10.1111/1753-0407.70177)
Supplement: Supplementary file 1 — Table S1: Stratified characteristics according to depressive symptoms status. Table S2: Stratified characteristics according to type II diabetes status. [file JDB-18-e70177-s001.docx]

***Type 2 Diabetes and Factors Associated with Depressive Symptoms in Mexico: A Cross-Sectional Analysis of 11,913 Individuals in the 2022 National ENSANUT Survey***

**This supplementary material accompanies the main article and provides additional data tables, figures, and methodological details that support the findings presented in the manuscript.**

**Table S1:** Stratified characteristics according to depressive symptoms status

|  | ‡No or mild depressive symptoms (%) | weighted % | ‡Moderate or severe depressive symptoms (%) | weighted% |
| --- | --- | --- | --- | --- |
| Diabetes |  |  |  |  |
| no | 8565 (82.8) | (85.3) | 1786 (17.3) | (14.7) |
| yes | 1006 (65.2) | (67.2) | 538 (34.8) | (32.8) |
| Sex |  |  |  |  |
| Males | 4121 (87.2) | (88.6) | 603 (12.8) | (11.4) |
| Females | 5465 (76.0) | (78.6) | 1724 (24.0) | (21.5) |
| Age |  |  |  |  |
| 20-59 | 7928 (87.1) | (88.7) | 1178 (12.9) | (11.3) |
| 60+ | 1658 (59.1) | (61.7) | 1149 (40.9) | (38.3) |
| Education |  |  |  |  |
| below secondary | 398 (57.7) | (56.5) | 292 (42.3) | (43.6) |
| Secondary school | 2211 (69.9) | (73.4) | 952 (30.1) | (26.6) |
| High school or above | 6977 (86.6) | (88.3) | 1083 (13.4) | (11.8) |
| Well-being index |  |  |  |  |
| Low | 2987 (75.9) | (78.7) | 947 (24.1) | (21.3) |
| Medium | 3206 (80.5) | (83.4) | 777 (19.5) | (16.6) |
| High | 3393 (84.9) | (87.0) | 603 (15.1) | (13.0) |
| Area |  |  |  |  |
| Urban | 7318 (81.1) | (84.2) | 1710 (18.9) | (15.9) |
| Rural | 2268 (78.6) | (80.1) | 617 (21.4) | (19.9) |
| Region |  |  |  |  |
| Pacific North | 1119 (82.8) | (84.9) | 232 (17.2) | (15.1) |
| Border | 1768 (81.3) | (83.8) | 407 (18.7) | (16.2) |
| Pacific-central | 579 (78.8) | (81.3) | 156 (21.2) | (18.7) |
| North centre | 2162 (79.3) | (82.0) | 566 (20.8) | (18.0) |
| Centre | 682 (82.3) | (84.6) | 147 (17.7) | (15.4) |
| Edo Mexico | 1003 (79.2) | (83.5) | 264 (20.8) | (16.5) |
| South pacific | 1015 (81.6) | (84.7) | 229 (18.4) | (15.3) |
| Peninsula | 1258 (79.4) | (82.0) | 326 (20.6) | (18.0) |
| Total | 9586 (80.5) | (83.3) | 2327 (19.5) | (16.7) |

‡ Depressive Symptoms score calculated based on CESD-7 scale was used.; for the adults aged between 20 and 59 is greater than 9 and greater than 5 for those aged 60 or more

**Table S2:** Stratified characteristics according to type II diabetes status

|  | No type II diabetes (%) | weighted % | Type II diabetes (%) | weighted% |
| --- | --- | --- | --- | --- |
| †‡ Depression (by age group) |  |  |  |  |
| no | 8565 (89.5) | (91.2) | 1006 (10.5) | (8.8) |
| yes-(age 20-59) | 1008 (85.8) | (88.0) | 167 (14.2) | (12.0) |
| yes- (age 60+) | 778 (67.7) | (67.6) | 371 (32.3) | (32.4) |
| Sex |  |  |  |  |
| Males | 4184 (88.6) | (89.8) | 540 (11.4) | (10.2) |
| Females | 6167 (86) | (88.5) | 1004 (14.0) | (11.5) |
| Age |  |  |  |  |
| 20-59 | 8331 (91.7) | (93.0) | 757 (8.3) | (7.0) |
| 60+ | 2020 (72) | (73.9) | 787 (28.0) | (26.2) |
| Education |  |  |  |  |
| below secondary | 519 (75.2) | (76.4) | 171 (24.8) | (23.6) |
| Secondary school | 2525 (79.9) | (82.7) | 635 (20.1) | (17.3) |
| High school or above | 7307 (90.8) | (92.0) | 738 (9.2) | (8.0) |
| Well-being index |  |  |  |  |
| Low | 3440 (87.5) | (89.7) | 490 (12.5) | (10.3) |
| Medium | 3452 (86.8) | (89.3) | 524 (13.2) | (10.7) |
| High | 3459 (86.7) | (88.6) | 530 (13.3) | (11.4) |
| Area |  |  |  |  |
| Urban | 7824 (86.8) | (89.0) | 1187 (13.2) | (11.0) |
| Rural | 2527 (87.6) | (89.8) | 357 (12.4) | (10.3) |
| Region |  |  |  |  |
| Pacific North | 1198 (88.8) | (90.1) | 151 (11.2) | (9.9) |
| Border | 1865 (86) | (88.5) | 303 (14.0) | (11.5) |
| Pacific-central | 649 (88.3) | (90.5) | 86 (11.7) | (9.6) |
| North centre | 2353 (86.4) | (88.5) | 371 (13.6) | (11.5) |
| Centre | 729 (87.9) | (88.0) | 100 (12.1) | (12.0) |
| Edo Mexico | 1097 (86.8) | (89.5) | 167 (13.2) | (10.5) |
| South pacific | 1088 (87.5) | (88.9) | 155 (12.5) | (11.1) |
| Peninsula | 1372 (86.7) | (89.0) | 211 (13.3) | (11.0) |
| Total | 10351 (87) | (89.1) | 1544 (13.0) | (10.9) |

†based on CESD-7 scale

‡ Depressive Symptoms score calculated based on CESD-7 scale was used.; for the adults aged between 20 and 59 is greater than 9 and greater than 5 for those aged 60 or more
